# Gait performance and prefrontal cortex activation during single and dual task walking in older adults with different cognitive levels

**DOI:** 10.3389/fnagi.2023.1177082

**Published:** 2023-06-02

**Authors:** Wei-Han Weng, Yea-Ru Yang, Nai-Chen Yeh, Pei-Hsin Ku, Po-Shan Wang, Ying-Yi Liao, Ray-Yau Wang

**Affiliations:** ^1^Department of Physical Therapy and Assistive Technology, National Yang Ming Chiao Tung University, Taipei, Taiwan; ^2^Department of Neurology, Taipei Municipal Gan-Dau Hospital, Taipei, Taiwan; ^3^Department of Gerontological Health Care, National Taipei University of Nursing and Health Sciences, Taipei, Taiwan

**Keywords:** mild cognitive impairment, dementia, cognitive function, motor function, brain activity

## Abstract

**Background:**

Growing evidence shows the cognitive function influences the motor performance. The prefrontal cortex (PFC) as a part of the executive locomotor pathway is also important for cognitive function. This study investigated the differences in motor function and brain activity among older adults with different cognitive levels, and examined the significance of cognition on motor functions.

**Methods:**

Normal control (NC), individuals with mild cognitive impairment (MCI) or mild dementia (MD) were enrolled in this study. All participants received a comprehensive assessment including cognitive function, motor function, PFC activity during walking, and fear of fall. The assessment of cognitive function included general cognition, attention, executive function, memory, and visuo-spatial. The assessment of motor function included timed up and go (TUG) test, single walking (SW), and cognitive dual task walking (CDW).

**Results:**

Individuals with MD had worse SW, CDW and TUG performance as compared to individuals with MCI and NC. These gait and balance performance did not differ significantly between MCI and NC. Motor functions all correlated with general cognition, attention, executive function, memory, and visuo-spatial ability. Attention ability measured by trail making test A (TMT-A) was the best predictor for TUG and gait velocity. There were no significant differences in PFC activity among three groups. Nevertheless, the PFC activated more during CDW as compared with SW in individuals with MCI (*p* = 0.000), which was not demonstrated in the other two groups.

**Conclusion:**

MD demonstrated worse motor function as compared to NC and MCI. The greater PFC activity during CDW in MCI may be considered as a compensatory strategy for maintaining the gait performance. Motor function was related to the cognitive function, and the TMT A was the best predictor for the gait related performance in present study among older adults.

## 1. Introduction

Due to the rapid increase in worldwide population, aging has become a widely focused issue in the recent decade. Aging leads to a gradual decrease in physical capacity, such as slow walking speed and greater gait variability, besides, it also causes the decline of cognitive function (Salthouse, 2004). Cognitive function comprises several domains, including memory, language, attention, visuospatial, and executive function (Tangen et al., 2014; Harvey, 2019). During normal aging, decline in certain cognitive abilities are expected, such as decreased processing speed and reduced attention. However, these changes should be subtle and not result in impairment in daily functions (Harada et al., 2013). Dementia, is a neurodegenerative syndrome which leads to deterioration of cognitive function beyond expectations and significantly influences the independence of daily activities and quality of life in older adults (Simons et al., 2006). Between normal aging and dementia, there is an intermediate state called mild cognitive impairment (MCI). Individuals with MCI have cognitive decline greater than expected for their age and education level, but not yet meeting the diagnostic criteria for dementia (Petersen, 2009; Petersen et al., 2014). It is suggested that people with MCI tend to have higher risk of dementia than healthy older adults (Manly et al., 2008).

The control of gait and balance are typically thought to be automatic in adults. However, growing evidences demonstrated that people with cognitive impairment not only suffer from significant cognitive decline but also show worse balance and walking performances than cognitively intact older adults, suggesting cognition is involved in gait and balance control. According to previous studies, people with Alzheimer’s Disease (AD) showed worse balance control relative to normal control (NC) and MCI (Leandri et al., 2009; Tangen et al., 2014). People with MCI showed worse balance control relative to NC when eyes closed but not when eyes opened (Leandri et al., 2009). For gait performance, no differences were found between people with MCI, dementia, and healthy older adults during single walking (SW) condition in some studies (Muir et al., 2012; Ansai et al., 2017). However, other studies reported a significant slower velocity and greater stride length during SW condition in people with MCI and dementia as compare with NC (Bahureksa et al., 2017; Peel et al., 2019; Teo et al., 2021). On the other hand, it is believed that complex walking condition, such as dual-task walking (DTW), may enhance the gait impairments (Bahureksa et al., 2017; Bishnoi and Hernandez, 2021). In some studies, people with early-stage of dementia showed worse DTW performance relative to people with MCI and NC (Ansai et al., 2017; Åhman et al., 2020). However, Ansai et al. (2017) did not find significant differences between people with MCI and NC during DTW. Therefore, the significance of cognition on walking needs further exploration. Regarding the influence of different cognitive domains on motor function, previous study showed visuospatial domain was independently associated with the time up and go (TUG) test in people with MCI and AD (Ansai et al., 2018). Additionally, language and visuospatial domains can predict the 10-m walk test measure in people with MCI, AD, and NC (Ansai et al., 2018). Another study demonstrated that slow walking speed associated with low executive function in older people with MCI (Doi et al., 2014). Nevertheless, the influence of specific cognitive domains on DTW performance needs further elucidation.

The prefrontal cortex (PFC) is important for cognitive function, and is also a part of the executive locomotor pathway (Hamacher et al., 2015). A review indicated PFC showed greater activation during imagining walking and cognitively demanding walking (Holtzer et al., 2014). In recent years, functional near-infrared spectroscopy (fNIRS) has been widely used to evaluate changes in brain hemodynamic response during walking (Gramigna et al., 2017). Some studies demonstrated that older adults with greater PFC activity during SW as compared to young adults, and the PFC activity showed greater response to the dual task paradigms (Mirelman et al., 2017; Nóbrega-Sousa et al., 2020). In healthy older adults, greater frontal lobe activations during DTW compared to SW were associated with reduced grey matter volume (Wagshul et al., 2019). Therefore, the deficits in cognitive function may influence the PFC activations in response to different tasks. One study demonstrated that PFC activation during DTW is significantly lower in older adults with cognitive impairment than normal older adults (Nosaka et al., 2022). The results of Holtzer and Izzetoglu’s (2020) study indicated MCI was related to attenuated PFC activation pattern during DTW. On the other hand, Udina et al. (2022) revealed that people with MCI showed greater prefrontal cerebral blood flow from the SW to the DTW compared to NC. However, to our knowledge, no study has investigated the differences in PFC activity during SW and DTW among older adults with different cognitive levels.

In this study, we aimed to compare the walking performance and PFC activity during SW and DTW in older adults with different cognitive levels. We also examined the relationships between cognitive function in different domains and walking performances. We hypothesized that people with cognitive impairment may demonstrate worse motor function and lesser PFC activation relative to normal older adults. Executive function and attention will be the most significant cognitive domains to correlate with motor function.

## 2. Materials and methods

### 2.1. Participants

Older adults from local community centers were recruited between December 2020 to March 2022. The inclusion criteria for participants with mild dementia (MD) were (1) age ≥ 65 years old, (2) diagnosed with dementia per physician (Hugo and Ganguli, 2014), (3) Mini- Mental State Examination (MMSE) score between 20 and 26 (Wilcock et al., 2008; Scheltens et al., 2012; Ascher-Svanum et al., 2015), and (4) ability to walk at least for 1 min independently without an assistive device. The inclusion criteria for participants with MCI were (1) age ≥ 65 years old, (2) MCI was determined by MMSE ≥24 and MoCA (with academic permission) < 26 (Nasreddine et al., 2005; Liu-Ambrose et al., 2008; ten Brinke et al., 2015; Creavin et al., 2016), (3) no evidence of dementia, and (4) ability to walk at least for 1 min independently without an assistive device. The inclusion criteria for NC were (1) age ≥ 65 years old, (2) MMSE ≥24 without MCI or dementia, and (3) ability to walk at least for 1 min independently without an assistive device.

The exclusion criteria were (1) other medical diagnosis of neurological or psychiatric disorder, e.g., stroke, (2) severe uncorrected visual or auditory disorders, and (3) moderate or advanced dementia.

All participants were informed about the research procedures and signed a written consent form. The study protocol was approved by the Institutional Review Board of National Yang-Ming University and was registered prospectively at Thai Clinical Trial Registry (TCTR20210301003).

### 2.2. Study design and measurements

This was a cross-sectional study. Characteristic data including age, gender, height, weight, educational level, fall history, and MMSE score were obtained before the study measurement. The cognitive function, motor function, brain activity, and fear of fall were the measurements for group comparisons in this study.

#### 2.2.1. Cognitive function

##### 2.2.1.1. General cognition

General cognition: The Chinese version Alzheimer’s disease assessment scale–cognitive subscale (ADAS-cog) was used to assess general cognition. It consists of 12 items with score ranging from 0 to 75, and a higher score indicates the more dysfunction (Jiang et al., 2020).

##### 2.2.1.2. Executive function

The Frontal assessment battery (FAB) was used to assess the executive function. It is a short and easily administered cognitive test. FAB consists of six parts, including conceptualization, mental flexibility, programming, sensitivity to interference, inhibitory control and environment autonomy with a total score of 18. Higher scores indicate better function (Mok et al., 2004). The Chinese version trail making test-B (TMT-B) was also used to assess executive function especially the mental flexibility and set-shifting. During the test, participants drew lines in an ascending but alternating order between numbers and Chinese words (number 1–8 and Monday–Sunday in Chinese). This test has been used in our previous study to assess executive function in older adults with MCI (Kuo et al., 2022). The time needed to complete the test was recorded.

##### 2.2.1.3. Attention

The TMT-A was used to assess attention and processing speed (Tangen et al., 2014; Treviño et al., 2021). Participants drew lines to connect numbers from 1 to 25 in an ascending sequence during the test. The time needed to complete the test was recorded.

##### 2.2.1.4. Memory

The Chinese version verbal learning test (CVVLT) consisting 9 two-character nouns presented over four learning trials was used to assess memory both immediately and after 10-min delay (Chang et al., 2010). The total number of nouns correctly remembered in any order was recorded.

##### 2.2.1.5. Visuo-spatial ability

The clock drawing test (CDT) was used to evaluate the visuo-spatial ability. Participants draw the numbers on the face of the clock, and then draw the hands to show a specific time “10 after 11” (Sadeghipour Roodsari et al., 2013). We used the 10-point scoring system to score CDT (Manos and Wu, 1994).

#### 2.2.2. Motor function

##### 2.2.2.1. Balance and mobility

TUG test was used to measure the balance and mobility. Participants were asked to perform the task “quickly, but safely,” and the time needed to complete the test was recorded (Nordin et al., 2006). The average of 2 trials was used for analysis.

##### 2.2.2.2. Gait performance

The gait performance was assessed under SW and cognitive dual task walking (CDW) conditions 3 times for each walking condition (a total of six walking trials with random order). Participants walked on the GAITRite walkway back and forth for 60 s during each walking condition with a 60-s rest in between. During SW condition, the participants were asked to walk at their comfortable speed. During CDW, the participants walked at their comfortable speed while subtracting 3 from an initial three-digit number serially. The gait parameters in this study included velocity (cm/s), cadence (step/min), stride length (cm), and gait variability (%). Gait variability is defined as the coefficient of variation (= standard deviation / mean × 100%) of stride time (Yogev et al., 2005; Dubost et al., 2006). Besides, the dual task cost (DTC) of gait velocity was calculated to quantify the interference of cognitive task on walking performance. The following formula was used for the DTC calculation: (cognitive dual task walking velocity – single walking velocity) / single walking velocity x 100% (Plummer and Eskes, 2015). The more negative value of DTC indicates the more interference of the additional task.

#### 2.2.3. Brain activity

**Figure 1:**
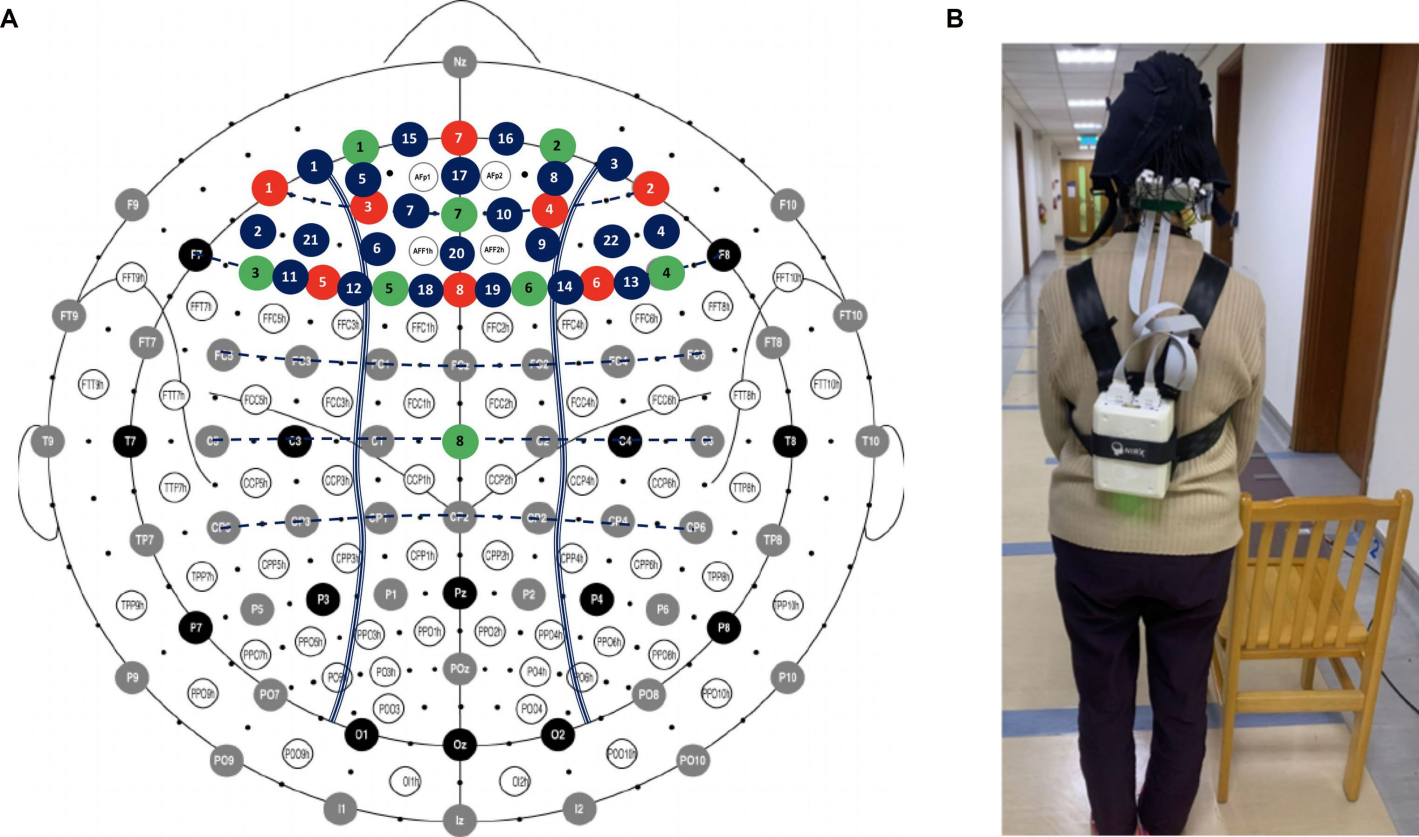
Arrangement of the fNIRS optodes **(A)** Locations of sources, detectors, and channels **(B)** Participants carry a fNIRS control box with straps during each of the walking condition. Red: source; Green: detector; Blue: channel.

Brain activity during each walking condition was measured by a dual-wavelength (760 and 850 nm) multi-channel wearable functional near-infrared spectroscopy (fNIRS) imaging system (NIRSport2, NIRx Medical Technologies LLC, Glen Head, NY, United States). A total of 22 source-detector (8 LED sources and 7 detectors) channels were placed on participant’s head to assess the hemodynamics of PFC (Figure 1A). We used an overcap to avoid interference of ambience light during the experiment. Before measurement, a calibration was conducted to evaluate the amplification factor and quality of signal. The quality of signal indicated by fNIRS software should reach “excellent” or at least “acceptable” to proceed the measurement.

To ensure the quality of the data, serial preprocessing was conducted. The relative coefficient of variation (CV, in %) for channel and trial at each wavelength were calculated (CVchannel and CVtrial). CVchannel >15% and CVtrial >10% were used as the standards to remove the channels and trials. The remaining signals were band-filtered (low-cutoff frequency 0.005 Hz, high-cutoff frequency 0.03 Hz) (Piper et al., 2014), and wavelet filtering was used to correct the motion artifacts in each channel (Molavi and Dumont, 2012). Next, the preprocessed signals were converted to concentration in oxygenated (HbO) and deoxygenated hemoglobin (HbR) by the modified Beer–Lambert law (Cope and Delpy, 1988; Boas et al., 2001; Kocsis et al., 2006). A 5-s baseline collected before each trial was used to obtain the relative changes in HbO and HbR concentrations for walking condition. The changes of HbO and HbR between 5 to 40 s during walking were averaged over three repetitions for each walking condition. The hemoglobin differential (Hbdiff = HbO – HbR) were calculated to indicate the PFC activity, and were used for data analysis (Lu et al., 2015). The fNIRS signals were processed using the HOMER2 package. The calculation of CV and Hbdiff values were performed using home-made scripts developed on MATLAB (Mathworks, Natick, MA, United States). The way of participants carry a fNIRS control box with straps during walking is demonstrated in Figure 1B.

#### 2.2.4. Fear of fall

The fear of fall was measured by modified falls efficacy scale (MFES) which is a 14 items questionnaire. Each item scores from 0 to 10, and higher scores reflect more confidence and less fear of falling (Hill et al., 1996).

### 2.3. Statistical analysis

SPSS version 24.0 for Windows (SPSS Inc., Chicago, IL, United States) was used to analyze the collected data. Kolmogorov–Smirnov test was used to test normal distribution. To explore the differences among groups, the one-way ANOVA was used for normally distributed variables (e.g., motor performances), Chi-square test was used for categorical data, and Kruskal-Wallis test were used for non-normally distributed variables (e.g., demographic data and cognition). Tukey test and Dunn-Bonferroni test were used to perform the post-hoc analysis. Two-way repeated-measures ANOVAs, with group and condition as factors, were used to assess the brain activity. For *post hoc* analysis, Bonferroni correction (with significance level correction: 0.05/number of comparisons) was used. The Spearman’s rank correlation coefficient was used to assess the associations between motor function and cognition. Linear multiple regression (stepwise method) was further used to verify if the different domain of cognitive function could predict the motor performance. The statistically significant level was set at *p* < 0.05.

### 2.4. Sample size calculation

The sample size was calculated using the G^*^Power 3.1 software. A minimum of 42 subjects were necessary, with type I error at 5%, statistical power at 80%, and effect size of 0.5 (medium effect size). Considering 20% of possible missing data, we recruited 51 participants for this study.

## 3. Results

There were fifty-one (41 female and 10 male) participants in the study, including 17 participants with MD, 17 participants with MCI, and 17 participants as NC. Demographic information and cognitive function of the participants are presented in Table 1. There were no significant differences among groups for age, gender, education, and history of falls. The median age for all participants was 71 (interquartile range, IQR = 75–69) years old. The people with MD demonstrated significant lower MMSE score than MCI group (*p* = 0.01) and NC group (*p* = 0.000).

**Table 1:** Demographic information and cognitive function of the participants.

	Group			*p* value
	Normal control (*n* = 17)	Mild cognitive impairment (*n* = 17)	Mild dementia (*n* = 17)	
Age (years)	70 (68.5, 73.5)	71 (70, 75.5)	71 (69.3, 77.3)	0.489
Gender (F/M)	(16/1)	(13/4)	(12/5)	0.172
Education (years)	12 (12, 16)	12 (9, 14)	12 (6.8, 12)	0.060
Fall history in recent 6 months	0 (0, 0)	0 (0, 0)	0 (0, 0)	0.829
MMSE	28 (26.5, 28.5)	26 (25, 28)	24 (22.3, 26)^*#^	0.000
ADAS-cog	5 (3.7, 9.8)	11.3 (9.5, 13.2)^*^	14.8 (11.4, 20.6)^*^	0.000
FAB	16 (14.5, 17)	13 (12, 16.5)^*^	13 (11.3, 15)^*^	0.001
TMT A (sec)	35.3 (28.8, 40.3)	45.6 (30.7, 57.5)	52.1 (44.5, 82.8)^*^	0.003
TMT B- Chinese version (sec)	27.2 (21.6, 40.9)	41.1 (27.6, 56.2)	49.7 (44.7, 89.5)^*^	0.001
CVVLT 30 s (number)	8 (7, 9)	7 (5, 8)	5 (5, 6)^*#^	0.000
CVVLT 10 min (number)	7 (4.5, 8)	5 (3.5, 6.5)	1 (0.3, 4)^*#^	0.000
CDT	10 (10, 10)	10 (9.5, 10)	7.5 (2.5, 10)^*#^	0.000

Values are median (interquartile) with Kruskal-Wallis test. Mini-Mental State Examination, MMSE; Alzheimer’s Disease Assessment Scale-cognitive subscale, ADAS-cog; Frontal assessment battery, FAB; Trail making test, TMT; Chinese version verbal learning test, CVVLT; Clock drawing test, CDT. ^*^p < 0.05 vs. Normal control; ^#^ *p* < 0.05 vs. MCI.

### 3.1. Cognitive function

Participants with MD demonstrated significant lower scores on ADAS-cog (*p* = 0.000), FAB (*p* = 0.001), TMT A (*p* = 0.002), and TMT B (*p* = 0.001) than the NC group. The MD group also showed significant lower scores on CVVLT 30s (*p* = 0.023 vs. MCI group, *p* = 0.000 vs. NC group), CVVLT 10 min (*p* = 0.003 vs. MCI group, *p* = 0.000 vs. NC group), and CDT (*p* = 0.009 vs. MCI group, p = 0.000 vs. NC group) as compared with MCI group and NC group. In addition, participants with MCI showed significant lower score on ADAS-cog (*p* = 0.024) and FAB (*p* = 0.034) than the NC (Table 1).

### 3.2. Motor function

Table 2 shows the motor function in three groups. There were no significant differences between the NC group and MCI group in TUG, SW, and CDW performances. Participants with MD showed significant greater time of TUG test (*p* = 0.001), slower SW velocity (*p* = 0.023), slower CDW velocity (*p* = 0.002), lesser cadence (*p* = 0.026) and stride length (*p* = 0.045), and greater in DTC (*p* = 0.039) compared with the NC. Participants with MD also showed significant greater time of TUG test (*p* = 0.012), slower SW velocity (*p* = 0.04), lesser cadence (*p* = 0.02), and slower CDW velocity (*p* = 0.04) compared with participants with MCI.

**Table 2:** Comparisons of motor functions and fear of fall among groups.

	Group				*p* value		
	Normal control (*n* = 17)	Mild Cognitive Impairment (*n* = 17)	Mild dementia (*n* = 17)	*F*; *p* value	*p* value normal vs. MCI	*p* value normal vs. dementia	*p* value MCI vs. dementia
TUG (sec)	7.61 ± 1.82	8.18 ± 1.07	10.00 ± 2.18^*#^	8.379; 0.001	0.612	0.001	0.012
MFES	8.63 ± 1.24	8.72 ± 0.63	8.45 ± 0.90	0.343; 0.712	0.964	0.844	0.697
SW – velocity (cm/s)	120.68 ± 22.27	119.14 ± 14.75	102.13 ± 20.63^*#^	4.571; 0.015	0.971	0.023	0.040
SW – cadence (step/min)	117.78 ± 13.32	119.71 ± 8.83	108.73 ± 11.16^#^	4.431; 0.017	0.871	0.065	0.020
SW – stride length (cm)	121.53 ± 13.76	119.84 ± 11.52	113.58 ± 19.62	1.234; 0.300	0.944	0.302	0.471
SW – gait variability (%)	2.44 ± 1.54	2.59 ± 1.17	3.31 ± 3.47	0.679; 0.512	0.980	0.522	0.638
CDW – velocity (cm/s)	105.4 ± 22.97	97.83 ± 19.99	79.84 ± 18.17^*#^	6.703; 0.003	0.533	0.002	0.040
CDW – cadence (step/min)	110.02 ± 13.15	107.75 ± 12.51	98.08 ± 12.63^*^	4.034; 0.024	0.862	0.026	0.086
CDW – stride length (cm)	114.29 ± 15.09	109.04 ± 13.91	99.24 ± 22.58^*^	3.126; 0.053	0.659	0.045	0.252
CDW – gait variability (%)	3.72 ± 2.87	6.80 ± 7.65	5.38 ± 3.42	1.522; 0.229	0.204	0.715	0.648
Dual task cost (%)	−12.77 ± 9.59	−18.37 ± 9.8	−21.73 ± 8.80^*^	3.828; 0.029	0.206	0.039	0.671

Values are mean ± standard deviation with One way ANOVA. Timed up and go, TUG; Modified falls efficacy scale international, MFES; Single walking, SW; Cognitive dual task walking, CDW. ^*^p < 0.05 vs. Normal control; ^#^*p* < 0.05 vs. MCI.

### 3.3. Correlations between cognitive function and motor function

The correlations between different domains of cognitive function and motor function are presented in Table 3. The time needed to complete TUG test was significantly correlated with ADAS-cog, FAB, TMT-A, TMT-B, CVVLT, and CDT (rho = −0.474 – 0.522). The velocity of SW was significantly correlated with ADAS-cog, TMT-A, TMT-B, CVVLT, and CDT (rho = −0.505 – 0.454). The cadence of SW was significantly correlated with ADAS-cog, TMT-A, TMT-B, and CVVLT (rho = −0.506 – 0.421). Stride length was significantly correlated with TMT-B (rho = −0.304). As for CDW, the velocity was significantly correlated with ADAS-cog, FAB, TMT-A, TMT-B, CVVLT, and CDT (rho = −0.592 – 0.467). The cadence of CDW was significantly correlated with ADAS-cog, TMT-A, TMT-B, and CVVLT (rho = −0.508 – 0.336) and stride length was significantly correlated with FAB, TMT-A, TMT-B, CVVLT 30s, and CDT (rho = −0.424 – 0.364). Gait variability of CDW was significantly correlated with FAB, TMT-A, and TMT-B (rho = −0.478 – 0.434).

**Table 3:** Correlations between different domains of cognition and motor performances.

	ADAS- cog	FAB	TMT- A	TMT- B	CVVLT 30s	CVVLT 10 min	CDT
TUG (sec)	0.473^**^	−0.290^*^	0.522^**^	0.502^**^	−0.474^**^	−0.351^*^	−0.295^*^
SW – velocity (cm/s)	−0.367^**^	0.258	−0.498^**^	−0.505^**^	0.454^**^	0.304^*^	0.335^*^
SW – cadence (step/min)	−0.287^*^	0.181	−0.480^**^	−0.506^**^	0.421^**^	0.364^**^	0.147
SW – stride length (cm)	−0.184	0.211	−0.274	−0.304^*^	0.272	0.100	0.271
SW – gait variability (%)	−0.009	−0.120	0.091	0.053	−0.012	−0.112	−0.119
CDW – velocity (cm/s)	−0.417^**^	0.414^**^	−0.592^**^	−0.591^**^	0.467^**^	0.334^*^	0.408^**^
CDW – cadence (step/min)	−0.286^*^	0.269	−0.508^**^	−0.484^**^	0.336^*^	0.326^*^	0.161
CDW – stride length (cm)	−0.267	0.357^*^	−0.360^*^	−0.424^**^	0.328^*^	0.176	0.364^**^
CDW – gait variability (%)	0.128	−0.478^**^	0.380^**^	0.434^**^	−0.227	−0.222	−0.161

Alzheimer’s Disease Assessment Scale-cognitive subscale, ADAS-cog; Frontal assessment battery, FAB; Trail making test, TMT; Chinese version verbal learning test, CVVLT; Clock drawing test, CDT; Timed up and go, TUG; Single walking, SW; Cognitive dual task walking, CDW. ^*,**^ *p* < 0.05, <0.01.

A further analysis by linear multiple regression showed that TMT-A was the best predictor of TUG performance (R^2^ = 0.43, *p* = 0.000). ADAS-cog also contributed to about 5% to explain the TUG performance (*R*^2^ = 0.057, *p* = 0.016). Furthermore, the regression analysis identified that TMT-A was the best predictor of both SW-velocity (*R*^2^ = 0.257, *p* = 0.000) and CDW-velocity (R^2^ = 0.324, *p* = 0.000).

### 3.4. Fear of fall

There was no significant difference between groups in the results of MFES (Table 2).

### 3.5. PFC activation

**Figure 2:**
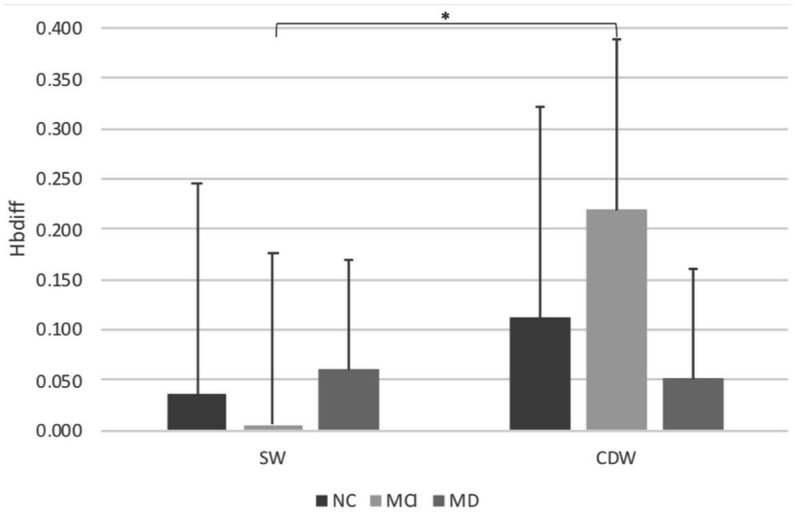
Brain activation of PFC indicated by Hbdiff during different walking conditions. Bar graphs represent Hbdiff levels. Single walking, SW; Cognitive dual task walking, CDW; Normal control, NC; Mild cognitive impairment, MCI; Mild dementia, MD. *Indicates significant differences.

The results of brain activation of PFC during different walking conditions in different groups are presented in Table 4 and Figure 2. Significant group and condition interactions were observed (*p* = 0.021). *Post hoc* tests revealed intragroup differences for people with MCI. People with MCI showed greater PFC activation during CDW compared to SW (p = 0.000). However, no significant differences were observed in NC (*p* = 0.318) or people with MD (*p* = 0.823) between SW and CDW. Regarding between-group differences, no significance was observed during both SW and CDW. No significant group main effect was revealed among groups. Significant condition main effect was revealed (*p* = 0.004).

**Table 4:** Brain activation of PFC indicated by Hbdiff during different walking conditions.

	Group			*p* value		
	Normal control (*n* = 17)	Mild cognitive impairment (*n* = 17)	Mild dementia (*n* = 17)	Group	Condition	Interaction
SW	0.035 ± 0.21	0.006 ± 0.17	0.060 ± 0.11	0.561	0.004	0.021
CDW	0.112 ± 0.30	0.219 ± 0.13	0.052 ± 0.13			

Values are mean ± standard deviation. Single walking, SW; Cognitive dual task walking, CDW.

## 4. Discussion

In this study, we found people with MD demonstrated worse SW, CDW and TUG performance as compared to people with MCI and NC. However, such gait-related performance did not differ significantly between people with MCI and NC. These motor functions correlated with general cognition, attention, executive function, memory, and visuo-spatial ability, except SW performance did not correlate significantly with the FAB. We further noted that the attention ability measured by TMT-A was the best predictor for TUG, SW and CDW performance, especially the velocity. Regarding PFC activity during SW or CDW, there were no significant differences between people with MD and NC or between people with MCI and NC. But the PFC activated more during CDW as compared with SW in people with MCI, which was not demonstrated in people with MD or NC.

In our study, we found deteriorations of both SW and CDW ability in individuals with MD. Such findings had also been demonstrated in previous studies (Gras et al., 2015; Teo et al., 2021). The changes of gait parameters such as slower velocity occur alongside with the progression of cognitive decline. When the cognitive resources are reduced in people with cognitive impairment, the changes of motor performances appear (Seidler et al., 2010). Slow walking speed is suggested as an indicator of cognitive dysfunction since its association with poor psychomotor function and attention (Sui et al., 2020). However, Muir et al. (2012) and Ansai et al. (2017) reported worse dual task walking performance but not SW performance in people with MD as compared with normal older adults. The SW performance was measured by continuous walking for 1 min in present study to indicate the functional walking ability, while Muir et al. and Ansai et al. measured the SW ability with relatively short distance (6 m in Muir’s study and 12.4 m in Ansai’s study). Therefore, it may indicate people with mild dementia demonstrate insufficient walking performance only under long walking distance. It should be noted that some other factors, such as age and severity of the cognitive impairments, may also affect the gait performances in people with MD. On the other hand, dual task paradigms have been widely used in neurodegenerative disease to evaluate the cognitive-motor relationship. Performing cognitive and motor two tasks concurrently will lead to greater utility of cognitive resources than either task performed alone. According to a longitudinal study, alterations of dual task gait function may be more sensitive than SW performance to predict the degeneration of cognitive functions, and be used in early diagnosis of dementia (Montero-Odasso et al., 2017).

Our results also demonstrated lack of differences in SW and CDW performance between people with MCI and NC, which was also noted in other study (Ansai et al., 2017). However, one meta-analysis reported an association between gait speed and cognitive function by a meaningful reduction of SW speed of 0.11 m/s in people with MCI compared with NC (Peel et al., 2019). Another meta-analysis also indicated that gait velocity and stride length discriminated best between MCI and healthy controls under SW condition. Moreover, dual-task assessment increased the discriminative power of gait variables (Bahureksa et al., 2017). Regarding such different findings, the characteristics of enrolled participants may be one of the possible reasons. Kyrdalen et al. (2019) reported that gait performance was associated with educational level, fall history, depressive symptoms, and number of medications in older adults. In our study, most of the baseline data of MCI group were comparable with NC group. Therefore, we speculated that people with relatively high educational level and good health condition may be able to preserve their motor function even though they were experiencing certain decline in cognitive function. In addition, types of secondary cognitive task may also influence the discrimination power of cognitive status in older adults. Previous studies suggested that the more complex cognitive tasks such as naming animals, the more deterioration in gait functions (Beauchet et al., 2005; Montero-Odasso et al., 2012; Muir et al., 2012). The relatively simple subtraction 3 from a three-digit number was chosen in present study due to this cognitive activity is similar to daily activities frequently performed. Therefore, people with MCI as in our study can still cope with most of the daily cognitive dual-task walking.

TUG test is an easy and reliable measure for quantifying functional mobility and balance ability, and is also used to screen fall risk in older adults (Podsiadlo and Richardson, 1991; Asai et al., 2018). Ten seconds is considered as a cut-off point for fall risks by TUG test (Bohannon, 2006). Similar to other results (Eggermont et al., 2010; Gras et al., 2015), we also noted a significant greater time needed to complete the TUG test in people with MD as compared with people with MCI and NC. We further noted 8 of the 17 (47%) participants needed more than 10 s to complete the test, while no one needed more than 10 s to complete the test in MCI or NC group. Regarding people with MCI, no difference was found relative to NC which was consistent with previous studies on TUG test (Eggermont et al., 2010; Ansai et al., 2017). Therefore, it should be noted that people with relatively severe cognitive impairment, such as MD may have higher fall risk.

According the relationships between motor and cognitive function, the TUG, SW, and CDW performance were all significantly correlated with general cognitive status and function in different cognitive domains in our study, indicating the interactions between the two systems. Among these relationships, the attention and executive function correlated moderately with gait-related performances. Previous studies also noted the importance of attention and executive function for balance control and walking performance in older adults (Woollacott and Shumway-Cook, 2002; Tangen et al., 2014). It is noted that different types of the walking tasks would highlight the importance of different cognitive domains. In present study, we found TMT-A indicating the attention ability, was the best predictor for TUG, SW velocity, and CDW velocity. However, the motor tasks tested in our study were considered relatively low task loads for older adults. On the other hand, Ansai et al. (2018) found visuo-spatial ability was highly associated with dual task TUG performance in older people with cognitive impairment.

Although we did not demonstrate the significant difference of PFC activation among people with different cognitive levels during either SW or CDW, Tomioka et al. (2009) demonstrated less prefrontal cortex activities during simulated driving in people with dementia than normal control. Another recent article noted greater left PFC activity during SW and lesser left PFC activity during CDW in people with dementia as compared with healthy older adults (Teo et al., 2021). Based on the compensation-related utilization of neural circuits hypothesis (CRUNCH), increased brain activity during simple task in people with cognitive impairment is a way for maintaining performance. However, increasing task demands may recruit the cognitive resources close to the ceiling, and lead to insufficient neural processing and decreased performance (Reuter-Lorenz and Cappell, 2008). Therefore, task complexity might be one of the possible reasons for the different results between our study and previous study. The other reason may be the age and severity of cognitive impairment. In Teo et al.’s (2021) study, participants were with older age (86.1 ± 7.3 years old) and more severe dementia than participants in present study. Thus, neural inefficiency-related phenomenon might be easier to be observed in people with relatively old age and advanced dementia.

However, it draws our attention that the brain activity was significantly more during CDW than during SW in people with MCI. This result was in line with Doi et al.’s (2013) and Udina et al.’s (2022) findings in people with MCI, and Teo et al.’s (2021) findings in people with subjective memory complains. Previously, we observed older adults without cognitive impairment did not activate more PFC, but activated more in supplementary motor area and premotor cortex during CDW as compared with SW (Lu et al., 2015). We thus speculate that people with MCI may need to activate more brain activities, including the PFC, to maintain their gait performance comparable to normal older adults during CDW. Such greater PFC activity was not shown in people with MD. Our result suggests that possible neural compensation seems to be impaired in people with MD due to more severe neurodegeneration.

There are some limitations should be noted in this study. First, the sample size of present study was relatively small, and the significance of this study could be strengthened by a larger sample size. In addition, family-wise error rate may be inflated due to multiple measures in the small samples. Second, we only measured the gait performance and brain activities during dual task walking; however, the performance of cognitive task during dual task walking may provide additional information. Third, we only measured PFC activity in this study. Other areas such as the motor or supplementary motor cortices may also change during motor tasks, and such changes need to be explored in future studies. Fourth, the task priority was not emphasized during the CDW in present study. The different task priorities during CDW may influence the task performance and brain activity. Fifth, the Chinese version TMT-B used in present study contains less number to be paired which may influence our results on predictive power. Finally, MCI is characterized as amnestic MCI (aMCI) because of small sample size in present study, we did not provide the subtype analysis.

## 5. Conclusion

The gait performance was related to the cognitive function, and people with MD demonstrated significant gait impairments as compared to normal older adults and people with MCI. People with MCI were able to maintain certain gait performance, and the greater PFC activity during dual task walking may be considered as a compensatory strategy for maintaining the performance. Our results also showed that attention ability indicated by TMT A was the best predictor for the gait related performance in older people across different cognitive functions.

## Data availability statement

The raw data supporting the conclusions of this article will be made available by the authors, without undue reservation.

## Ethics statement

The studies involving human participants were reviewed and approved by Institutional Review Board of National Yang-Ming University. The patients/participants provided their written informed consent to participate in this study.

## Author contributions

W-HW, Y-RY, and R-YW designed the study. W-HW, N-CY, and P-HK performed the experiments, analyzed the data and interpreted the data. W-HW wrote the first draft of the manuscript and edited the manuscript. P-SW confirmed the medical diagnosis of subjects. Y-YL, Y-RY, and R-YW provided critical review of the manuscript. All authors contributed to the article and approved the submitted version.

## Funding

This study is supported by Ministry of Science and Technology (MOST 110-2314-B-A49A-509 -MY2) and National Health Research Institutes (NHRI-EX112-10913PI).

## Conflict of interest

The authors declare that the research was conducted in the absence of any commercial or financial relationships that could be construed as a potential conflict of interest.

## Publisher’s note

All claims expressed in this article are solely those of the authors and do not necessarily represent those of their affiliated organizations, or those of the publisher, the editors and the reviewers. Any product that may be evaluated in this article, or claim that may be made by its manufacturer, is not guaranteed or endorsed by the publisher.

## Figures and Tables

**Table:** 

MCI	Mild cognitive impairment
AD	Alzheimer’s Disease
NC	Normal control
SW	Single walking
DTW	Dual-task walking
TUG	Time up and go
PFC	Prefrontal cortex
fNIRS	Functional near-infrared spectroscopy
MD	Mild dementia
MMSE	Mini- Mental State Examination
ADAS-cog	Alzheimer’s disease assessment scale–cognitive subscale
FAB	Frontal assessment battery
TMT	Trail making test
CVVLT	Chinese version verbal learning test
CDT	Clock drawing test
CDW	Cognitive dual task walking
DTC	Dual task cost
CV	Coefficient of variation
HbO	oxygenated hemoglobin
HbR	Deoxygenated hemoglobin
MFES	Modified falls efficacy scale
CRUNCH	Compensation-related utilization of neural circuits hypothesis
